# The associations between VDR BsmI polymorphisms and risk of vitamin D deficiency, obesity and insulin resistance in adolescents residing in a tropical country

**DOI:** 10.1371/journal.pone.0178695

**Published:** 2017-06-15

**Authors:** Rayinda Rahmadhani, Nur Lisa Zaharan, Zahurin Mohamed, Foong Ming Moy, Muhammad Yazid Jalaludin

**Affiliations:** 1 Department of Pharmacology, Faculty of Medicine, University of Malaya, Kuala Lumpur, Malaysia; 2 Julius Centre University of Malaya, Department of Social and Preventive Medicine, Faculty of Medicine, University of Malaya, Kuala Lumpur, Malaysia; 3 Department of Paediatrics, Faculty of Medicine, University of Malaya, Kuala Lumpur, Malaysia; Third Military Medical University Daping Hospital and Research Institute of Surgery, CHINA

## Abstract

**Background:**

The vitamin D receptor (VDR) gene is expressed abundantly in different tissues; including adipocytes and pancreatic beta cells. The rs1544410 or BsmI single nucleotide polymorphism (SNP) in the intronic region of the VDR gene has been previously associated with vitamin D levels, obesity and insulin resistance.

**Aims:**

This study was aimed to examine the association between BsmI polymorphism and risk of vitamin D deficiency, obesity and insulin resistance in adolescents living in a tropical country.

**Methods:**

Thirteen-year-old adolescents were recruited via multistage sampling from twenty-three randomly selected schools across the city of Kuala Lumpur, Malaysia (n = 941). Anthropometric measurements were obtained. Obesity was defined as body mass index higher than the 95^th^ percentile of the WHO chart. Levels of fasting serum vitamin D (25-hydroxyvitamin D (25(OH)D)), glucose and insulin were measured. HOMA-IR was calculated as an indicator for insulin resistance. Genotyping was performed using the Sequenom MassARRAY platform (n = 807). The associations between BsmI and vitamin D, anthropometric parameters and HOMA-IR were examined using analysis of covariance and logistic regression.

**Result:**

Those with AA genotype of BsmI had significantly lower levels of 25(OH)D (*p* = 0.001) compared to other genotypes. No significant differences was found across genotypes for obesity parameters. The AA genotype was associated with higher risk of vitamin D deficiency (*p* = 0.03) and insulin resistance (*p* = 0.03) compared to GG. The A allele was significantly associated with increased risk of vitamin D deficiency compared to G allele (adjusted odds ratio (OR) = 1.63 (95% Confidence Interval (CI) 1.03–2.59, *p* = 0.04). In those with concurrent vitamin D deficiency, having an A allele significantly increased their risk of having insulin resistance compared to G allele (adjusted OR = 2.66 (95% CI 1.36–5.19, *p* = 0.004).

**Conclusion:**

VDR BsmI polymorphism was significantly associated with vitamin D deficiency and insulin resistance, but not with obesity in this population.

## Introduction

The vitamin D endocrine system takes part in various biological processes including musculoskeletal development, erythropoiesis and blood pressure regulation [1]. Yet, it was estimated that one billion people had deficient or insufficient levels of vitamin D based on population studies [2]. The prevalence of vitamin D deficiency varies between different regions of the world whilst showing seasonal variations [3–7]. Factors that have been associated with increased risk of vitamin D deficiency include low skin exposure to sunlight, low dietary intake of vitamin D, high body mass index (BMI), and genetic predispositions [8–12].

Interestingly, approximately two thirds of adults in Malaysia, a country in South East Asia, had vitamin D deficiency despite receiving sunlight all-year round (it is located at 3 degree north of the equator) [13]. More worryingly, a study on thirteen-year old Malaysian adolescents revealed that nearly eighty percent of them fulfilled the criteria for vitamin D deficiency [14]. In both Malaysian adults and adolescents, adiposity was associated with vitamin D deficiency [13, 14]. Furthermore, the prevalence of obesity among children and adolescents in this country was also rather disturbing, being the highest in the South East Asian region with boys at 22% and girls at 19% [15]. Studies that examined factors that may contribute to the relationship between vitamin D levels and obesity are therefore required to further understand the possible mechanisms that may link them both, especially in our young population.

In addition to environmental contributions, genetic factors may account for 23% to 80% of variability in serum vitamin D levels, as observed in twin studies [16, 17]. There is, however, currently a dearth in studies that examined possible genetic contributors to vitamin D deficiency in the Malaysian population. Amongst single nucleotide polymorphisms (SNP) that may be associated with vitamin D levels are those located on the vitamin D receptor (VDR) gene (18). The VDR gene plays a crucial role in the modulation of vitamin D pathways and regulation of hormone responsive genes [18]. Interestingly, the VDR gene is also expressed in adipocytes and pancreatic beta cells and thus may influence body composition by directly regulating the differentiation and metabolism of adipocytes; or indirectly by insulin modulation [19, 20]. One of the SNPs in the VDR gene associated with vitamin D levels is the rs1544410 SNP, located in the intronic region (intron 8 near the 3´end). This SNP is a restriction fragment length polymorphism of the restriction endonucleases BsmI [21] and is also commonly known as the BsmI polymorphism. It is thought to affect VDR translational activity due to its strong linkage disequilibrium with a polyadenosine (poly (A)) microsatellite repeat in the 3´untranslated region [22]. In addition to vitamin D levels, the BsmI polymorphism has also been shown to be associated with obesity, insulin resistance and type 2 diabetes in some population [23–25]. Unfortunately, very few studies examined the relative influence of this polymorphism on vitamin D levels, adiposity and insulin resistance in the paediatric population [26–28]. Thus, this cross-sectional study was aimed to examine the association between BsmI (rs1544410) VDR gene polymorphism with vitamin D deficiency, adiposity and insulin resistance in our adolescents.

## Methodology

### Study participants

Ethical approval for this study was obtained from the Ethics Committee of the University of Malaya Medical Centre (MEC 896.123). The permission to carry out this study was obtained from the Ministry of Education and the principals of the respective participating schools. Thirteen-year-old participants were recruited via multistage sampling from 23 randomly selected government-funded secondary schools across the city of Kuala Lumpur, Malaysia from January 2012 to July 2012. Written informed consent was obtained by the researchers from either parents or guardians of each of the participants prior to the study.

### Anthropometric and pubertal assessments

Anthropometric measurements were taken by trained researchers following a standard protocol. Body weight of the participants was measured with participants in light clothing and shoes removed using a digital calibrated floor scale (SECA 813; Seca GmbH&Co., Hamburg, Germany) to the nearest 0.1kg. Height with shoes removed was measured using a portable stadiometer (SECA 813; Seca GmbH&Co., Hamburg, Germany) to the nearest 0.1cm. BMI was calculated as baseline body weight divided by height squared (kg/m^2^). Waist circumference (WC) was measured at the midway between iliac crest and the 10^th^ rib, according to WHO STEPS protocol [29]. Hip circumference (HC) was measured at the widest extension of the buttocks. Both WC and HC were measured using inelastic measurement tape (SECA 203, Hamburg, Germany). Waist-Hip Ratio (WHR) was calculated as WC divided by HC (WC/HC). Body fat percentage (BF%) was measured using a validated portable body composition analyzer (Inbody 230; Biospace Co.Ltd, Seoul, Korea) according to the instruction manual provided. Pubertal stage was self-assessed using a coloured Tanner stages illustration [30].

### Vitamin D level measurement

Fasting venous blood (after at least 8 hours of fasting) was drawn from the participants by trained physicians in a sitting position between 8 am to 9 am in the morning. Vitamin D levels (25-hydroxyvitamin D (25(OH)D)) were determined by an accredited clinical diagnostic laboratory (CDL) at the University of Malaya Medical Centre (UMMC), Kuala Lumpur, Malaysia adhering to standard clinical laboratory protocols. The kits used were Elecsys® Vitamin D total assay (Cobas, Roche Diagnostics Limited, Switzerland) which implements an electro-chemiluminescence binding assay (ECLIA) to determine the total 25(OH)D in-vitro. Measurements were conducted according to manufacturer’s protocol.

### Measures of insulin resistance

Fasting blood glucose (FBG) and fasting insulin (FI) were measured by the clinical diagnostic laboratory as mentioned above. Insulin resistance was calculated using a homeostatic model assessment of insulin resistance (HOMA-IR); HOMA-IR = FI (μU/mL) x FBG (mmol/L)/ 22.5.

### Genotyping

Genomic DNA from blood samples was extracted using QiAmp DNA Mini Kit (Qiagen, Hilden, Germany). The quality of the extracted DNA was such that the absorbance ratios of at least 1.8 was attained for both 260/280 and 260/230 readings. All DNA samples and duplicates were diluted to 10ng/μL and 20ng/μL, respectively, before being transferred to the respective wells. Quality controls included a blank and five duplicates. The VDR SNP of rs1544410 (BsmI) was genotyped using a Sequenom MassARRAY platform with iPLEX GOLD chemistry (Sequenom, San Diego, California) following the manufacturer’s protocols. The MassARRAY system was created based on the technology of MALDI-TOF (matrix-assisted laser desorption/ionization time-of-flight) mass spectrometry. The genotyping call rate was >95% for the variant.

### Statistical analyses

There were 941 participants, all of which had complete anthropometric data. Vitamin D levels were available in 678 participants, fasting insulin for 795 participants and genotyping for 807 participants. Obesity was defined as BMI higher than the 95^th^ percentile according to the WHO 2007 Growth Reference Data for 5–19 years [31]. Vitamin D status was categorized into three groups according to Misra *et al* [10] whereby vitamin D deficiency was defined as those with a 25(OH)-D level of ≤15 ng/mL (≤37.5 nmol/L), vitamin D insufficiency as those with a 25(OH)-D level between 15 ng/mL and 20 ng/mL (37.5–50 nmol/L) and vitamin D sufficiency as those with a 25(OH)-D level of ≥20 ng/mL (≥50 nmol/L). Insulin resistance was categorised based on HOMA-IR values as proposed by Yin *et al* [32], in which different cut-offs were used based on pubertal stages. A HOMA-IR >2.6 was used to define insulin resistance in pre-pubertal adolescents, while for pubertal adolescents the cut-off was a HOMA-IR >3.2.

Continuous variables were presented as means with standard deviations. All variables were tested for normal distribution using the Kolmogorov-Smirnov test. The Mann-Whitney U test was used to compare demographic and anthropometric measurements, vitamin D levels and insulin resistance between boys and girls. Categorical variables were presented as frequency and percentages and comparisons between genders were performed using chi-square testing. Correlation analyses were performed using Pearson’s correlation to examine the relationship between vitamin D levels and BMI as well as fasting insulin. In addition, binary logistic regression was used to examine the risk of obesity and insulin resistance in those with vitamin D deficiency compared to those with sufficient levels of vitamin D. Gender, ethnicities, maternal education and pubertal stage (and BMI for insulin resistance) were included as covariates. Maternal education was used as a proxy for socio-economic status [33].

Genotype distribution was assessed for Hardy-Weinberg equilibrium (HWE) by using the χ^2^ test. A *p*-value of more than 0.05 signifies agreement with HWE. Comparisons of adiposity parameters, 25(OH)D levels and metabolic status between genotypes (GG, GA and AA) were made using an analysis of covariance (ANCOVA) general linear model. The associations between VDR BsmI polymorphism (both genotypes GG, GA and AA as well as alleles G and A) and risk of vitamin D deficiency, obesity and insulin resistance were examined using binary logistic regression; adjusting for covariates mentioned above. In addition, the risks of obesity and insulin resistance as stratified according to vitamin D status comparing A allele (mutant) to the G allele (wild) were examined using binary logistic regression. A *p-*value of less than 0.05 was considered as statistically significant. Statistical analysis was performed using SPSS 24.0 software (IBM SPSS Statistics).

## Results

### Participants’ demographics, anthropometric data, vitamin D status and measures of insulin resistance

Overall, there were 941 (13 year old) participants, of which the majority were girls (72%) and of Malay ethnicity (75%) as presented in Table 1. The prevalence of obesity in our adolescent population was 21%, with 19% of girls and 29% of boys were categorised as obese while the prevalence of morbid obesity was 11%. The proportion of those considered as having insulin resistance was similar to obesity at 21%, with no significant differences found between boys and girls (20% vs 21% respectively). Of the 648 participants with 25(OH)D levels, 45% of them fulfilled the criteria for vitamin D deficiency (12% boys and 56% girls).

**Table 1:** Demographic, anthropometric and clinical profiles of thirteen-year old Malaysian adolescents in comparing boys and girls.

Characteristics			All participants	Boys	Girls	*p*-value
			n (%) or mean±SD	n (%) or mean±SD	n (%) or mean±SD	
N			941	261 (28%)	680 (72%)	
Ethnicity			941	261	680	0.003
	Malay		702 (75%)	210 (80%)	492 (72%)	0.003
	Chinese		121 (13%)	19 (7%)	102 (15%)	
	Indian		94 (10%)	29 (11%)	65 (10%)	
	Others		24 (2%)	3 (2%)	21 (3%)	
Pubertal status			928	258	670	0.03
	Pubertal		835 (90%)	188 (73%)	647 (97%)	
	Prepubertal		93 (10%)	70 (27%)	23 (3%)	
Maternal education			626	258	670	0.32
	Primary		58 (9%)	17 (11%)	41 (9%)	
	Secondary		415 (66%)	109 (69%)	306 (65%)	
	Tertiary		153 (24%)	32 (20%)	121 (26%)	
Weight (kg)			47.8 ±14.1	48.4 ±16.4	47.5±13.2	0.64
Height (cm)			151.5 ±7.5	151.4±9.6	151.6±6.5	0.82
BMI (kg/m^2^)			20.6 ±5.1	20.7±5.7	20.5±4.9	0.46
BMI categories			941	261	680	<0.0001
	Under-or Normal Weight		629 (67%)	154 (59%)	475 (70%)	
	Overweight		104 (11%)	30 (11%)	74 (11%)	
	Obese		208 (22%)	77 (29%)	131 (19%)	
Waist circumference (cm)			69.0 ±12.2	72.6±14.2	67.6±11.0	<0.0001
Hip circumference (cm)			85.3 ±11.6	86.1±12.3	85.0±11.4	0.713
Waist-Hip Ratio			0.81 ±0.25	0.84±0.01	0.80±0.01	<0.0001
Body fat percentage (BF%)			29.6 ±10.7	26.7±12.7	30.7±9.6	<0.0001
Vitamin D levels (ng/mL) (n = 678)			16.9±7.1	22.1±6.7	15.1±6.3	<0.0001
Vitamin D categories (n = 678)			678	174	504	<0.0001
		Sufficient	207 (30%)	102 (58%)	105 (21%)	
		Insufficient	166 (25%)	51 (29%)	115 (23%)	
		Deficient	305 (45%)	21(12%)	284 (56	
Fasting blood glucose (mmol/L)			4.7 ±0.4	4.7±0.4	4.6±0.4	0.116
Fasting insulin (μU/mL) (n = 795)			14.7 ±12.4	13.8±13.8	15.1±11.9	<0.0001
HOMA-IR (n = 795)			3.1 ±2.7	2.9±2.7	3.1±2.7	0.002
Insulin resistance (n = 795)			795	215	580	
		Normal	628 (79%)	171 (80%)	457 (79%)	0.43
		Insulin resistant	167 (21%)	44 (20%)	123 (21%)	

The majority of girls had already reached puberty compared to boys (*p* = 0.03). Comparing girls and boys in terms of adiposity parameters, boys have significantly higher waist circumferences and waist hip ratios while girls have significantly higher percentages of body fat (*p*<0.0001). In terms of biochemistry profiles, girls were found to have significantly lower 25(OH)D levels (*p*<0.0001) with higher levels of fasting insulin (*p*<0.0001) as compared to boys (Table 1).

The 25(OH)D levels were negatively correlated with both BMI (r = -0.130, *p* = 0.002) and fasting insulin (r = -0.127, *p* = 0.02), albeit weakly. Those with vitamin D deficiency was associated with increased risk of being obese (adjusted OR = 3.12, 95% CI 1.37–7.11, *p* = 0.007) but not of insulin resistance (adjusted OR = 1.78, 95% CI 0.80–3.97, *p* = 0.16) compared to those with sufficient levels after adjusting for covariates such as gender, ethnicity, maternal education, puberty status and BMI (for insulin resistance).

### The association between BsmI polymorphism and anthropometric measurements, vitamin D levels and measure of insulin resistance

The frequencies of GG, GA and AA genotypes of VDR BsmI SNP in our adolescent population met the criteria for the Hardy Weinberg Equilibrium. Those with a GG genotype had significantly higher levels of 25(OH)D, while those with the AA genotype had higher levels of fasting insulin and HOMA-IR values as compared to other genotypes (Table 2). However, after adjustment of covariates, there was no significant differences between genotypes in terms of fasting insulin and HOMA-IR. In addition, no significant differences in adiposity parameters were found across the three genotypes.

**Table 2:** Anthropometric measurements, vitamin D Levels and measures of insulin resistance according to rs1544410 (BsmI) genotypes.

Parameters	rs1544410 (n = 807)			*p*-value	Adjusted *p*-value^Φ^
	GG (n = 535)	GA (n = 232)	AA (n = 40		
Weight (kg)	47.6±13.8	48.1±13.8	50.1±20.3	0.79	0.18
Height (cm)	151.3±7.1	151.9±8.0	151.6±8.6	0.60	0.64
BMI (kg/m^2^)	20.6±5.0	20.6±5.1	21.8±7.5	0.91	0.26
Waist Circumference (cm)	68.9±11.8	69.4±12.3	72.6±16.7	0.56	0.16
Hip Circumference (cm)	85.2±11.3	85.8±11.1	85.7±18.7	0.90	0.49
Waist-Hip Ratio	0.8±0.3	0.8±0.1	0.9±0.2	0.15	0.69
Body fat percentage (BF %)	29.3±10.5	30.1±11.4	31.9±12.3	0.48	0.31
Vitamin D levels (ng/mL) (n = 678)	17.0±7.5	16.1±6.9	11.8±5.1	**0.001**	**0.001**
Fasting blood glucose (mmol/L)	4.6±0.4	4.7±0.4	4.6±0.4	0.29	0.28
Fasting insulin (μU/mL) (n = 795)	14.5±14.2	15.1±9.8	16.9±9.8	**0.04**	0.21
HOMA-IR	3.0±3.1	3.2±2.1	3.4±1.9	**0.03**	0.16

^Φ^ Adjusted for gender, ethnicity, maternal education and pubertal stage.

Those with an AA genotype of VDR BsmI were significantly associated with increased risk of vitamin D deficiency compared to the GG genotype (OR = 8.37 (95% CI 1.07, 65.71)) as presented in Table 3. No significant association was observed between BsmI polymorphism and risk of obesity in our adolescents (Table 4). The AA genotype was also found to confer a higher risk of insulin resistance compared to GG (OR = 2.75 (95% CI 1.13, 6.67)) as shown in Table 5.

**Table 3:** Association between VDR BsmI and risk of vitamin D deficiency in Malaysian adolescents presented as OR (unadjusted and adjusted) with 95% CI.

VDR rs1544410	Vitamin D Sufficiency (n = 162)	Vitamin D Deficiency (n = 272)	Unadjusted OR (95% CI)	*p*-value	Adjusted* OR (95% CI)	*p*-value
GG	119	180	Reference	-	-	-
GA	41	75	1.21 (0.78, 1.89)	0.40	1.26 (0.72, 2.21)	0.41
AA	2	17	5.62 (1.28, 24.77)	**0.02**	8.37 (1.07, 65.71)	**0.03**

*Adjusted for gender, ethnicity, pubertal status and maternal education.

**Table 4:** Association between VDR BsmI and risk of obesity in Malaysian adolescents presented as OR (unadjusted and adjusted) with 95% CI.

VDR rs1544410	Normal (n = 535)	Obese (n = 183)	Unadjusted OR (95% CI)	*p*-value	Adjusted* OR (95% CI)	*p*-value
GG	357	115	Reference	-	-	-
GA	152	56	1.14 (0.79, 1.66)	0.48	1.44 (0.77, 1.91)	0.42
AA	26	12	1.43 (0.70, 2.93)	0.32	1.21 (0.60, 3.46)	0.40

*Adjusted for gender, ethnicity, pubertal status and maternal education.

**Table 5:** Association between VDR BsmI and risk of insulin resistance in Malaysian adolescents presented as OR (unadjusted and adjusted) with 95% CI.

VDR rs1544410	Normal (n = 432)	Insulin Resistant (n = 248)	Unadjusted OR (95% CI)	*p*-value	Adjusted* OR (95% CI)	*p*-value
GG	297	158	Reference	-	-	-
GA	118	72	1.22 (0.81, 1.63)	0.44	1.16 (0.73, 1.83)	0.52
AA	17	18	2.00 (1.00, 3.97)	0.05	2.75 (1.13, 6.67)	**0.03**

*Adjusted for gender, ethnicity, pubertal status, maternal education and obesity.

Having the A allele of the VDR BsmI was significantly associated with increased risk of vitamin D deficiency in the Malaysian adolescent compared to the G allele (adjusted OR = 1.63 (95% CI 1.03, 2.59, *p* = 0.04) as presented in Table 6. No significant increased risk of obesity and insulin resistance was found with the A allele compared to the G allele in this population. However, when stratified according to vitamin D status, it was shown that in those with vitamin D deficiency, having an A allele significantly increased their risk of having insulin resistance compared to G allele (adjusted OR = 2.66 (95% CI 1.36, 5.19, *p* = 0.004) (Table 7).

**Table 6:** Risk of vitamin D deficiency, obesity and insulin resistance with A allele of VDR BsmI compared to G allele in Malaysian Adolescent Population presented as OR (unadjusted and adjusted) with 95% CI.

	G allele	A allele	Unadjusted OR (95% CI)	*p*-value	Adjusted OR (95% CI)	*p*-value
	Normal/cases	Normal/cases				
Vitamin D deficiency	279/435	45/109	1.51 (1.04, 2.18)	**0.02**	1.63 (1.03, 2.59) ∞	**0.04**
Obesity	866/286	204/80	1.17 (0.89, 1.55)	0.27	1.21 (0.86, 1.70) ∞	0.28
Insulin Resistance	712/388	152/108	1.27 (0.98, 1.66)	0.07	1.36 (0.99, 1.89) *	0.06

∞Adjusted for gender, ethnicity, maternal education and puberty stage.

*Adjusted for gender, ethnicity, maternal education, puberty stage and BMI.

**Table 7:** Risk of obesity and insulin resistance with A allele of VDR BsmI polymorphism compared to G allele in Malaysian adolescents when stratified according to Vitamin D status presented as adjusted OR with 95% CI.

	Vitamin D sufficiency				Vitamin D deficiency			
	G allele	A allele	Adjusted OR (95% CI)	*p*-value	G allele	A allele	Adjusted OR (95% CI)	*p*-value
	Normal/ cases	Normal/ cases			Normal/ cases	Normal/ cases		
Obesity	201/36	31/10	1.73 (0.58, 5.10) ∞	0.32	279/117	67/33	1.34 (0.75, 2.37)∞	0.32
Insulin Resistance	199/78	27/18	1.17 (0.33, 4.00) *	0.81	258/160	50/54	2.66 (1.36,5.19)*	**0.004**

∞Adjusted for gender, ethnicity, maternal education and puberty stage.

*Adjusted for gender, ethnicity, maternal education, puberty stage and BMI.

## Discussion

Results of this study indicated that more than a fifth of Malaysian adolescents in the capital city of Kuala Lumpur, were obese. More worryingly, a tenth were considered morbidly obese while a fifth were demonstrated to have insulin resistance. The percentage of those with vitamin D deficiency was forty-five percent with girls found to have significantly lower 25(OH)D levels compared to boys. We found that adolescents carrying the AA genotype of VDR BsmI were associated with increased risk of both vitamin D deficiency and insulin resistance compared to the GG genotype. The A allele of the BsmI was associated with significantly higher risk of vitamin D deficiency compared to the G allele. Moreover, in those with concurrent vitamin D deficiency, having an A allele was significantly associated with increased risk of insulin resistance in this population. Although vitamin D deficiency was associated with increased risk of obesity, we were not able to demonstrate any significant association between BsmI polymorphisms and adiposity parameters or risk of obesity in our adolescent population.

The prevalence of obesity in our study was comparable to the recent multi-country study by Marie Ng et al [15], although higher than the 9% prevalence documented from the Malaysian Health and Adolescents Longitudinal Research Team study (MyHeARTs) involving Malaysian adolescents of similar age [34]. The participants for our study were recruited from the urban city of Kuala Lumpur whilst those of MyHeARTs were recruited from three different states in Malaysia, including both urban and rural areas [34]. This study may be the first to examine insulin resistance in an adolescent population in Malaysia. Although the previously reported prevalence of metabolic syndrome was rather low at 3% in our adolescents [35], our findings on insulin resistance are of public health concern. More studies on insulin resistance in adolescents of this country are required to examine possible contributors, both environmental and genetic factors, as it can lead to increasing number of younger patients with type 2 diabetes mellitus. The proportion of adolescents with vitamin D deficiency, were much lower than that found in the MyHeARTs (78%) [14]. The discrepancy may be due to geographical differences as mentioned above. In addition, different assays were used to measure 25(OH)D levels and thus may contribute to the variability. Higher occurrence of vitamin D deficiency in our adolescent girls as compared to boys was similar to findings in primary-school children (7–12 years old) [36] and those of MyHeARTs [14]. Nevertheless, vitamin D deficiency in this population was still comparatively higher than other populations of children and adolescents from different regions in the world; for example, Denmark at 8% [4], Mexico at 18% [6] and Turkey at 25% during summer [37]. Possible contributors to vitamin D deficiency in the Malaysian population have been discussed in great depth by Sadat et al [14].

We demonstrated that those with AA (mutant) genotype of the BsmI had the lowest levels of vitamin D compared to other genotypes and had higher risk of vitamin D deficiency compared to the GG genotype (wild). The A allele itself was significantly associated with vitamin D deficiency in our population. Our finding is consistent with studies on Brazilian children where BsmI polymorphism was found to be associated with serum vitamin D level [26, 28] although it was the wild variant (GG) that was associated with lower vitamin D levels in Brazilian girls [26]. In contrast, the association between BsmI polymorphism and vitamin D levels was not supported in other populations such as the Arabs (Saudi) [38], Uygur and Kazakhs [39] as well as the European adolescents [40], suggesting that this SNP may influence vitamin D levels rather differently in different population.

The allele of the BsmI polymorphism is located at the intronic region of the VDR gene (intron 8) and near the 3’ end of the VDR gene and is thought to possibly alter the expression of other genes’ by affecting the stability of VDR mRNA and its gene transcription [41]. Interestingly, Ogunkolade et al demonstrated that although VDR polymorphisms were significant determinant of VDR mRNa and VDR protein levels in peripheral blood, there was no correlation found between VDR polymorphisms and vitamin D levels. Possible contribution of other VDR genes as well as interaction with environmental factors such as dietary supplements and physical activities needs to be further investigated to fully understand the role of this SNP in vitamin D deficiency in our population. It is worth noting that besides vitamin D levels, this SNP was also associated with calcium absorption especially in younger women [42]. However, a recent meta-analysis did not demonstrate the risk of osteoporosis with BsmI in postmenopausal women [43]. Hence, the role of this SNP in modulating vitamin D levels and its consequences on bone health need to be further elucidated in our population with a longitudinal study design.

As there were no significant differences found when comparing fasting insulin and HOMA-IR across the genotypes after adjustment of covariates, it is possible that other factors such as gender (as shown in the results), ethnicity [44], pubertal stages [32], and socioeconomic status [45] to also influence levels of insulin in this population. However, when insulin resistance was examined as a categorical variable with the cut-off for HOMA-IR chosen based on pubertal stage [32], it was found that the AA genotype of BsmI conferred higher risk of insulin resistance as compared to the GG genotype. The cohort of Brazilian children also reported to demonstrate association between this SNP and insulin resistance [28]. Thus, our study highlights the possible role of VDR BsmI polymorphism in mediating insulin resistance in early adolescence in non-Caucasian populations. In adults, similar findings on the relationship between BsmI and insulin resistance were reported in Caucasians [46] and Bangladeshi Asians [24] while studies on other population such as Brazilians [47], Egyptians and Polish women [48, 49] did not demonstrate significant association. The pancreatic beta cell’s expression of VDR [19] supports the idea that polymorphisms in the VDR region such as the BsmI may exert genomic actions possibly influencing insulin secretion [19, 50]. Ogunkalade et al demonstrated that VDR expression did determine insulin secretory capacity in a functional study [51]. While direct action on the pancreas has been postulated, this SNP has not been demonstrated to be associated with an increased susceptibility to type 1 diabetes mellitus on its own [52]. Although studies on BsmI suggested an association with type 2 diabetes mellitus, a consequence of insulin resistance, this was not reflected by meta-analyses as many of these studies were of small sample sizes [53, 54]. As insulin resistance and type 2 diabetes often arises as a result of a complex interplay between genetic and lifestyle influences, larger population based studies need to be carefully designed to further evaluate the association between this SNP and early onset insulin resistance.

We found a significant association between the A allele of BsmI and risk of insulin resistance in those with concurrent vitamin D deficiency. This is interesting as vitamin D deficiency on its own was not found to be associated with increased risk of insulin resistance. The VDR gene may modify the relationship between vitamin D and insulin resistance [51]. Jain et al previously reported that in New Zealand, South Asian women responded differently in terms of insulin sensitivity when given vitamin D supplementation according to VDR gene polymorphism (FokI) [55]. It was suggested that genotyping of the VDR gene may predict response to vitamin D intervention to improve insulin sensitivity [55]. Thus, future clinical study incorporating the VDR polymorphisms and vitamin D intervention may be desirable in our population of young adolescents with insulin resistance.

We did not find any significant differences between the genotypes of BsmI in terms of adiposity parameters. Furthermore, no significant association was observed between this SNP and risk of obesity, even when stratified according to vitamin D status. This was supported from findings in other populations such as Polish females (post-menopausal) [49], non-Hispanic white US adults [56] and US females [57]. On the contrary, BsmI was significantly associated with obesity in other populations such as the Arabs [38], French Caucasians [58], Polish males [24] and Swedish females [59]. VDR was shown to play a critical role in mediating the inhibitory actions of vitamin D on adipogenesis [60]. The expression of VDR mRNA also changes during adipocyte differentiation [61]. It was hypothesized by Ochs-Balcom et al that linkage disequilibrium with other functional SNPs in the 3’ VDR region may explain the association of BsmI with obesity [57].

This study provides additional data on vitamin D deficiency, obesity and insulin resistance among an adolescent population in Southeast Asia, a cohort on which data is still currently lacking. We implemented direct measurements of anthropometric profiles by trained researchers, therefore reducing self-reporting bias. Even though the schools were selected randomly across different zones in Kuala Lumpur, Malaysia, girls may have been overrepresented possibly due to volunteer bias. Although Malaysia is made up of three major ethnicities; the Malays, Chinese and Indians, only a third of participants were Chinese or Indian, thus inter-ethnic genetic comparisons were not examined. We measured total 25(OH)D which may not truly reflect vitamin D status. It was demonstrated that although total 25(OH)D was different between Americans of African and European decent, the free serum 25(OH)D was comparable [11, 62]. Thus, vitamin D deficiency diagnosed solely on total 25(OH)D measurements may be misleading in certain populations. Hence, more studies are required in our population to establish our vitamin D status.

This study focused solely on the BsmI polymorphism. There are other well studied VDR gene polymorphisms such as rs731236 (TaqI), rs7975232 (ApaI) and rs10735810 (FokI) [18] which may influence vitamin D levels, adiposity and insulin resistance in this population. The FokI polymorphism has been associated with increased risk of type 2 diabetes in the Asian population [54] and hence the role of this SNP needs to be explored further. In addition, the interactions between the genotypes of BsmI and vitamin D deficiency with risk of obesity and insulin resistance were not able to be examined as the sample size was relatively small for this purpose to allow meaningful analysis.

In conclusion, the VDR BsmI polymorphism was significantly associated with vitamin D deficiency and insulin resistance, but not with obesity. Functional studies are needed in future to further characterize the contribution of this polymorphism to risk of vitamin D deficiency and metabolic dysregulation in the younger population.
